# Diabetes Self-care Activities and Their Relation with Glycemic Control in Patients Presenting to The Indus Hospital, Karachi

**DOI:** 10.7759/cureus.6297

**Published:** 2019-12-05

**Authors:** Ayesha A Hai, Sundus Iftikhar, Saba Latif, Fivzia Herekar, Muhammad Junaid Patel

**Affiliations:** 1 Internal Medicine, The Indus Hospital, Karachi, PAK; 2 Statistics, Indus Hospital Research Center, The Indus Hospital, Karachi, PAK; 3 Internal Medicine and Infectious Diseases, The Indus Hospital, Karachi, PAK

**Keywords:** diabetes-related self-care activities, glycemic control, self management, self care

## Abstract

Introduction

Nowadays, chronic conditions are increasing globally, stressing on self-management and patients' responsibility toward recognizing and resolving issues related to their illness. Diabetes is also a chronic illness, and diabetes-related self-care activities have been shown to be promising towards preventing its complications and achieving optimal glycemic control.

Objective

1) To assess the association between glycemic control and diabetes-related self-care activities

2) To evaluate the association of patients' sociodemographic characteristics with diabetes-related self-care activities

3) To examine the impact of patients' sociodemographic characteristics on glycemic control

Materials and methods

This cross-sectional study was conducted at The Indus Hospital Karachi from February 2019 to July 2019. A total of 288 patients of both genders, age ≥18 years, having type 2 diabetes mellitus with glycated hemoglobin (HbA1c) done within the last three months from the interview date were enrolled in the study using a non-probability consecutive sampling technique. Whereas patients not giving consent for participation in the study, ICU admitted patients, critically ill patients, pregnant women, comatose, patients with type 1 diabetes mellitus, Alzheimer’s disease, dementia, coexisting and chronic liver disease were excluded from the study.

Results

Majority of the patients were female (n=209; 72.6%) and had uncontrolled glycemic control (n=235; 81.6%). Furthermore, less than half of the patients had inadequate diabetes-related self-care activities (n=140; 48.6%). The Mean ± SD of age was 51.9±10.2 years. The significantly higher proportion of patients who have had a duration of illness and treatment ≥3 years had uncontrolled diabetes but adequate diabetes-related self-care activities. Moreover, there was no association between diabetes-related self-care activities and glycemic control.

Conclusion

There was no significant relationship between diabetes-related self-care activities and glycemic control. Moreover, a higher proportion of patients with a longer duration of diabetes (≥3 years) had poor glycemic control but adequate diabetes-related self-care activities.

## Introduction

Diabetes mellitus (DM) is one of the most common non-communicable diseases, a major public health problem, and the most important cause of premature illness and deaths worldwide [1-2]. Globally, 425 million people are suffering from this disease and Pakistan stands as second (7.028 million) amongst the Middle East and North Africa [3].

DM is known for its progressive nature, chronicity, and complexity and is known as an illness of self-management as it requires regular blood glucose monitoring [4]. There are various ways to monitor blood glucose levels amongst them Glycated hemoglobin (HbA1c) is considered as the most reliable method. Internationally, it is accepted as a gold standard biochemical indicator of average blood glucose concentrations over the preceding three months [5]. A reasonable goal for most adults is <7% according to recent American Diabetes Association (ADA) guidelines [6].

The increasing prevalence of DM depends on several factors, including a sedentary lifestyle. Good glycemic control in diabetic patients cannot only be achieved through good compliance with medication but also depends largely upon a regular routine, which includes a healthy diet, glucose monitoring, and exercise. Lack of physical activity is the major root cause of all chronic diseases. It has been observed that the elderly population does not comply with exercise leading to a high rate of diabetes-related complications [7-8].

Studies have shown that diabetes-related self-care activities improve glycemic control [4,9-10]. Moreover, just a lifestyle modification in poorly controlled diabetic patients can lead to a reduction in HbA1c up to 1% [11]. This leads to an approximately 25% reduction in microvascular complications such as diabetic retinopathy, cataract, neuropathy, nephropathy, heart failure, and amputations [12-14].

Thus, this makes diabetes treatment a two-pronged approach: 1) anti-diabetic treatment and 2) the self-care management behavior of diabetic patients. Therefore, it is necessary to uncover the patients’ self-care factors that are associated with good glycemic control in our population. This will help in the counseling and education of patients regarding the disease and its available management approaches; in particular, lifestyle modifications.

Hence, the present study was conducted with the aim to: 1) assess the association between glycemic control and diabetes-related self-care activities, 2) evaluate the possible association of patients' sociodemographic characteristics with diabetes-related self-care activities, and 3) examine the impact of patients' sociodemographic characteristics on glycemic control.

## Materials and methods

This cross-sectional study was conducted at The Indus Hospital Karachi, which is a free-of-cost tertiary care facility, from February 2019 to July 2019. A priori sample size was calculated through PASS software (Power and sample size version 11.0; NCSS LLC., Kaysville, Utah) using various diabetes-related self-care activities [15] (glucose management, dietary control, physical control, health-care use, sum scale) with the following assumptions: 80% power, 95% confidence interval. The highest sample size came out to be 288.

Patients of both genders, age ≥18 years, having type 2 diabetes mellitus, with glycated hemoglobin (HbA1c) done within the last three months from the interview date were enrolled in the study. Whereas for patients not giving consent for participation in the study, the intensive care unit (ICU) admitted patients, critically ill patients, pregnant women, comatose patients, patients with type 1 diabetes mellitus, Alzheimer's disease, dementia, and coexisting and chronic liver disease were excluded from the study. All the eligible patients were recruited from the outpatient and diabetic clinic using a non-probability consecutive sampling technique.

Sociodemographic details included participants’ age, gender, education, ethnicity, family history of diabetes and occurrence of diabetes-related complications, and clinical characteristics, including systolic and diastolic blood pressure, height (in cm), weight (in kg), and body mass index (kg/m^2^), were recorded on a pre-designed questionnaire.

Diabetes-related self-care activities were assessed using the Diabetes Self-Management Questionnaire (DMSQ) [15]. The Patient Health Questionnaire (PHQ) was used to assess depression in study participants [16-17].

Data analysis procedure

Data were entered in REDCap software (Research Electronic Data Capture v9.1.0; Vanderbilt University, Nashville, Tennessee) and analyzed using SPSS version 24.0 (Statistical Package for Social Sciences; IBM Corp., Armonk, New York). Mean ± SD/Median (interquartile range; IQR) was computed as appropriate for all the quantitative variables like age, DMSQ score, PHQ score, income, education, HbA1c, duration of diabetes, and duration of treatment. Frequency and percentage were computed for all the categorical variables like gender, comorbid, self-care behavior (inadequate: DSMQ score <6 and adequate: DSMQ score ≥6), glycemic control (adequate: HbA1c <7.0 and inadequate: HbA1c≥7.0), depression (minimum/none: PHQ score ≤4, mild to moderate: PHQ score 5 to <15, and severe: PHQ score ≥15).

The independent sample T-test/Mann-Whitney U test was applied, as appropriate, to assess the difference in DMSQ score, PHQ score, and various sociodemographic variables between glycemic control. The independent sample T-test/Mann-Whitney U test was applied, as appropriate, to assess the difference in HbA1c, PHQ score, and various sociodemographic variables between the self-care behavior. The chi-square test/Fisher exact test was applied, as appropriate, to assess the association of various categorical variables with self-care behavior and glycemic control. P-value <0.05 was considered statistically significant.

## Results

A total of 288 patients were enrolled in the study, out of which 209 (72.6%) were females. The mean ± SD of age was 51.9 ± 10.2 years (Table 1). The median (IQR) of body mass index (BMI), education, income, duration of diabetes, duration of treatment, and DMSQ score of all the patients was 28.7 (25.5-32.7), 5 (0-10), 20k (15k-25k), 5 (2-12), 10 (6-16), and 6.0 (5.2-6.9), respectively (Table 1), with no significant difference between patients with controlled and uncontrolled diabetes except duration of diabetes and treatment (Table 1). The majority of the patients had uncontrolled glycemic control (n=235; 81.6%). Results showed that a significantly higher proportion of patients who have had a duration of illness and treatment ≥3 years had uncontrolled diabetes in comparison to those who had a duration of illness and treatment <3 years (76.2% vs 23.8%, p=0.009; 64.3% vs 35.7%, p=0.011, Table 1).

**Table 1:** Association of various study participants' characteristics with glycemic control Min, minimum; Max, maximum; SD, standard deviation; IQR, interquartile range; BMI, body mass index; DSMQ, Diabetes Self-Management Questionnaire; PHQ, Patient Health Questionnaire; PKR, Pakistani Rupee

	Glycemic control		Overall	P-value
	Uncontrolled (≥7)	Controlled (<7)		
Gender				
Male	67 (28.5)	12 (22.6)	79 (27.4)	0.387^ⱡ^
Female	168 (71.5)	41 (77.4)	209 (72.6)	
Total	235 (100)	53 (100)	288 (100)	
Age (Years)				
Mean ± SD	51.8 ± 10.1	52.4 ± 10.4	51.9 ± 10.2	0.703^ɫ^
Min-Max	18-82	35-80	18-82	
Median (IQR)	51 (45-59)	50 (44.5-60)	51 (45-59)	
BMI				
Mean ± SD	29.1 ± 5.2	29.7 ± 5.4	29.2 ± 5.2	0.704^ꝉ^
Min-Max	15-47.6	21.3-47.9	15-47.9	
Median (IQR)	28.7 (25.4-32.7)	28.8 (25.7-32.7)	28.7 (25.5-32.7)	
BMI categories				
Underweight	2 (0.9)	0 (0)	2 (0.7)	0.793^ƪ^
Normal	20 (8.5)	4 (7.5)	24 (8.4)	
Overweight	28 (12)	4 (7.5)	32 (11.1)	
Obese	184 (78.6)	45 (84.9)	229 (79.8)	
Total	234 (100)	53 (100)	287 (100)	
Education (Years)				
Mean ± SD	5.1 ± 5.1	5.6 ± 5.6	5.2 ± 5.2	0.646^ꝉ^
Min-Max	0-16	0-18	0-18	
Median (IQR)	5 (0-10)	5 (0-10)	5 (0-10)	
Education categories				
<5 years	109 (46.4)	24 (45.3)	133 (46.2)	0.885^ⱡ^
≥5 years	126 (53.6)	29 (54.7)	155 (53.8)	
Total	235 (100)	53 (100)	288 (100)	
Income (PKR)				
Mean ± SD	21364.9 ± 9696.9	19968.8 ± 11847.1	21K ± 10K	0.292^ꝉ^
Min-Max	5k-60k	1k-50k	1K-60K	
Median (IQR)	20k (15k-25k)	17k (10k-27250)	20K (15K-25K)	
Income categories				
<20k	65 (43.9)	17 (53.1)	82 (45.6)	0.343^ⱡ^
≥20k	83 (56.1)	15 (46.9)	98 (54.4)	
Total	148 (100)	32 (100)	180 (100)	
Duration of Diabetes (years)				
Mean ± SD	8.6 ± 7.3	6.4 ± 7	8.2 ± 7.3	0.017^*ꝉ^
Min-Max	0.1-30	0.2-30	1mon-30y	
Median (IQR)	6 (3-14)	4 (1-10)	5 (2-12)	
Duration of diabetes categories				
<3 years	56 (23.8)	22 (41.5)	78 (27.1)	0.009^*ⱡ^
≥3 years	179 (76.2)	31 (58.5)	210 (72.9)	
Total	235 (100)	53 (100)	288 (100)	
Duration of diabetes treatment				
Mean ± SD	6 ± 6.1	4.4 ± 5.8	5.7 ± 6.1	0.014^*ꝉ^
Min-Max	0-30	0.1-25	1.6w-30y	
Median (IQR)	4 (1.4-8.3)	2 (0.9-5)	4 (1-8)	
Duration of diabetes treatment categories				
<3 years	84 (35.7)	29 (54.7)	113 (39.2)	0.011^*ⱡ^
≥3 years	151 (64.3)	24 (45.3)	175 (60.8)	
Total	235 (100)	53 (100)	288 (100)	
Medication for diabetes				
Oral only	141 (60.3)	36 (67.9)	177 (61.7)	0.380^*ⱡ^
Insulin only	45 (19.2)	6 (11.3)	51 (17.8)	
Both	48 (20.5)	11 (20.8)	59 (20.6)	
Total	234 (100)	53 (100)	287 (100)	
PHQ Score				
Mean ± SD	10.9 ± 6.7	10.7 ± 6.1	10.7 ± 6.2	0.863^ꝉ^
Min-Max	1-23	0-25	0-25	
Median (IQR)	10 (5-16.5)	10 (6-16)	10 (6-16)	
PHQ Catogories				
Minimal/none	43 (18.3)	10 (18.9)	53 (18.4)	0.927^ⱡ^
Mild-Mod	122 (51.9)	26 (49.1)	148 (51.4)	
Moderately severe to Severe	70 (29.8)	17 (32.1)	87 (30.2)	
Total	235 (100)	53 (100)	288 (100)	
DSMQ				
Mean ± SD	6 ± 1.1	6.3 ± 1.1	6.0 ± 1.1	0.206^ꝉ^
Min-Max	2.9-9.6	4.4-9.4	2.9-9.5	
Median (IQR)	6 (5.2-6.7)	6.3 (5.4-7.3)	6.0 (5.2-6.9)	
DSMQ categories				
Adequate	116 (49.4)	24 (45.3)	140 (48.6)	0.592^ⱡ^
Inadequate	119 (50.6)	29 (54.7)	148 (51.4)	
Total	235 (100)	53 (100)	288 (100)	
I check my blood sugar levels with care and attention				
Does not apply to me	1 (1.9)	9 (3.8)	10 (3.5)	0.432^ⱡ^
Applies to me to some degree	9 (17)	55 (23.4)	64 (22.2)	
Good understanding	43 (81.1)	171 (72.8)	214 (74.3)	
Total	53 (100)	235 (100)	288 (100)	
The food I choose to eat makes it easy to achieve optimal blood sugar levels.				
Does not apply to me	5 (9.4)	17 (7.2)	22 (7.6)	0.059^*ⱡ^
Applies to me to some degree	7 (13.2)	65 (27.7)	72 (25)	
Applies to me to a considerable degree	25 (47.2)	112 (47.7)	137 (47.6)	
Applies to me very much	16 (30.2)	41 (17.4)	57 (19.8)	
Total	53 (100)	235 (100)	288 (100)	
I keep all doctor's appointments recommended for my diabetes treatment.				
Does not apply to me	0 (0)	3 (1.3)	3 (1)	0.970^ƪ^
Applies to me to some degree	5 (9.4)	22 (9.4)	27 (9.4)	
Applies to me to a considerable degree	14 (26.4)	68 (28.9)	82 (28.5)	
Applies to me very much	34 (64.2)	142 (60.4)	176 (61.1)	
Total	53 (100)	235 (100)	288 (100)	
I take my diabetes medication (e. g. insulin, tablets) as prescribed.				
Does not apply to me	0 (0)	3 (1.3)	3 (1)	0.986^ƪ^
Applies to me to some degree	6 (11.3)	27 (11.5)	33 (11.5)	
Applies to me to a considerable degree	16 (30.2)	74 (31.5)	90 (31.3)	
Applies to me very much	31 (58.5)	131 (55.7)	162 (56.3)	
Total	53 (100)	235 (100)	288 (100)	
Occasionally I eat lots of sweets or other foods rich in carbohydrates.				
Does not apply to me	18 (34)	66 (28.1)	84 (29.2)	0.408^ⱡ^
Applies to me to some degree	23 (43.4)	99 (42.1)	122 (42.4)	
Applies to me to a considerable degree	5 (9.4)	44 (18.7)	49 (17)	
Applies to me very much	7 (13.2)	26 (11.1)	33 (11.5)	
Total	53 (100)	235 (100)	288 (100)	
I record my blood sugar levels regularly (or analyze the value chart with my blood glucose meter).				
Does not apply to me	2 (3.8)	16 (6.8)	18 (6.3)	0.368^ⱡ^
Applies to me to some degree	13 (24.5)	49 (20.9)	62 (21.5)	
Applies to me to a considerable degree	9 (17)	62 (26.4)	71 (24.7)	
Applies to me very much	29 (54.7)	108 (46)	137 (47.6)	
Total	53 (100)	235 (100)	288 (100)	
I tend to avoid diabetes-related doctor's appointments. (In the day and night).				
Does not apply to me	20 (37.7)	97 (41.3)	117 (40.6)	0.191^ⱡ^
Applies to me to some degree	11 (20.8)	41 (17.4)	52 (18.1)	
Applies to me to a considerable degree	9 (17)	63 (26.8)	72 (25)	
Applies to me very much	13 (24.5)	34 (14.5)	47 (16.3)	
Total	53 (100)	235 (100)	288 (100)	
Do regular physical activity to achieve optimal blood sugar levels.				
Does not apply to me	8 (15.1)	26 (11.1)	34 (11.8)	0.846^ⱡ^
Applies to me to some degree	13 (24.5)	57 (24.3)	70 (24.3)	
Applies to me to a considerable degree	14 (26.4)	62 (26.4)	76 (26.4)	
Applies to me very much	18 (34)	90 (38.3)	108 (37.5)	
Total	53 (100)	235 (100)	288 (100)	
I strictly follow the dietary recommendations given by my doctor or diabetes specialist.				
Does not apply to me	4 (7.5)	11 (4.7)	15 (5.2)	0.588^ⱡ^
Applies to me to some degree	10 (18.9)	63 (26.8)	73 (25.3)	
Applies to me to a considerable degree	18 (34)	73 (31.1)	91 (31.6)	
Applies to me very much	21 (39.6)	88 (37.4)	109 (37.8)	
Total	53 (100)	235 (100)	288 (100)	
I do not check my blood sugar levels frequently enough as would be required for achieving good blood glucose control.				
Does not apply to me	12 (22.6)	33 (14)	45 (15.6)	0.363^ⱡ^
Applies to me to some degree	19 (35.8)	83 (35.3)	102 (35.4)	
Applies to me to a considerable degree	15 (28.3)	72 (30.6)	87 (30.2)	
Applies to me very much	7 (13.2)	47 (20)	54 (18.8)	
Total	53 (100)	235 (100)	288 (100)	
I avoid physical activity, although it would improve my diabetes.				
Does not apply to me	13 (24.5)	56 (23.8)	69 (24)	0.970^ⱡ^
Applies to me to some degree	22 (41.5)	91 (38.7)	113 (39.2)	
Applies to me to a considerable degree	10 (18.9)	48 (20.4)	58 (20.1)	
Applies to me very much	8 (15.1)	40 (17)	48 (16.7)	
Total	53 (100)	235 (100)	288 (100)	
I tend to forget to take or skip my diabetes medication (e. g. insulin, tablets).				
Does not apply to me	20 (37.7)	58 (24.7)	78 (27.1)	0.196^ⱡ^
Applies to me to some degree	12 (22.6)	81 (34.5)	93 (32.3)	
Applies to me to a considerable degree	11 (20.8)	47 (20)	58 (20.1)	
Applies to me very much	10 (18.9)	49 (20.9)	59 (20.5)	
Total	53 (100)	235 (100)	288 (100)	
Sometimes I have real food binges (not triggered by hypoglycemia).				
Does not apply to me	12 (22.6)	52 (22.1)	64 (22.2)	0.954^ⱡ^
Applies to me to some degree	16 (30.2)	75 (31.9)	91 (31.6)	
Applies to me to a considerable degree	13 (24.5)	50 (21.3)	63 (21.9)	
Applies to me very much	12 (22.6)	58 (24.7)	70 (24.3)	
Total	53 (100)	235 (100)	288 (100)	
Regarding my diabetes care, I should see my medical practitioner(s) more often.				
Does not apply to me	5 (9.4)	20 (8.5)	25 (8.7)	0.910^ⱡ^
Applies to me to some degree	8 (15.1)	32 (13.6)	40 (13.9)	
Applies to me to a considerable degree	14 (26.4)	74 (31.5)	88 (30.6)	
Applies to me very much	26 (49.1)	109 (46.4)	135 (46.9)	
Total	53 (100)	235 (100)	288 (100)	
I tend to skip planned physical activity.				
Does not apply to me	12 (22.6)	55 (23.4)	67 (23.3)	0.926^ⱡ^
Applies to me to some degree	23 (43.4)	91 (38.7)	114 (39.6)	
Applies to me to a considerable degree	12 (22.6)	57 (24.3)	69 (24)	
Applies to me very much	6 (11.3)	32 (13.6)	38 (13.2)	
Total	53 (100)	235 (100)	288 (100)	
My diabetes self-care is poor.				
Does not apply to me	14 (26.4)	42 (17.9)	56 (19.4)	0.166^ⱡ^
Applies to me to some degree	20 (37.7)	77 (32.8)	97 (33.7)	
Applies to me to a considerable degree	8 (15.1)	68 (28.9)b	76 (26.4)	
Applies to me very much	11 (20.8)	48 (20.4)	59 (20.5)	
Total	53 (100)	235 (100)	288 (100)	
**P-value<0.0001, ꝉ Mann Witney U test, ɫ Independent sample T-test, ƪ Fisher-exact test, ⱡ Chi-square test				

Furthermore, less than half of the patients had inadequate diabetes-related self-care activities (n=140; 48.6%) with similar results as for glycemic control. No significant difference was observed in age, BMI, education, and HbA1c between the categories of diabetes-related self-care activities (Table 2). However, the duration of illness and treatment was found to be significantly associated with diabetes-related self-care activities. Results showed that a greater number of patients with longer duration of diabetes and its treatment (≥3 years) had more adequate diabetes-related self-care activities in comparison to others (78.4% vs 21.6%, p=0.032, 68.2% vs 31.8%, p=0.008, Table 2).

**Table 2:** Association of various study participants' characteristics with diabetes-related self-care activities Min, minimum; Max, maximum; SD, standard deviation; IQR, interquartile range; BMI, body mass index; HTN, hypertension; IHD, ischemic heart disease; CVA, cerebrovascular accident; DSMQ, Diabetes Self-Management Questionnaire; PHQ, Patient Health Questionnaire

	DSMQ Categories			
	Inadequate (<6)	Adequate (≥6)	Total n (%)	p.value
Gender				
Male	42 (30)	37 (25)	79 (27.4)	0.342^ǂ^
Female	98 (70)	111 (75)	209 (72.6)	
Total	140 (100)	148 (100)	288 (100)	
Age (Years)				
Mean ± SD	52.2 ± 10.4	51.6 ± 9.9	51.9 ± 10.2	0.713^ɫ^
Min-Max	18-82	21-76	18-82	
Median (IQR)	52 (45-59.8)	50 (46-59)	51 (45-59)	
BMI				
Mean ± SD	29.4 ± 5.5	29.1 ± 5	29.2 ± 5.2	0.807^ɫ^
Min-Max	15-47.9	16.4-45.9	15-47.9	
Median (IQR)	28.8 (25.4-32.6)	28.6 (25.6-32.7)	28.7 (25.5-32.7)	
BMI				
Underweight	1 (0.7)	1 (0.7)	2 (0.7)	0.975^ƪ^
Normal	11 (7.9)	13 (8.8)	24 (8.4)	
Overweight	16 (11.4)	16 (10.9)	32 (11.1)	
Obese	112 (80)	117 (79.6)	229 (79.8)	
Total	140 (100)	147 (100)	287 (100)	
Professional activity				
Employed	42 (30)	31 (20.9)	73 (25.3)	0.077^Ɨ^
Unemployed	98 (70)	117 (79.1)	215 (74.7)	
Total	140 (100)	148 (100)	288 (100)	
Education (Years)				
Mean ± SD	4.8 ± 5.3	5.6 ± 5.1	5.2 ± 5.2	0.172^ɫ^
Min-Max	0-16	0-18	0-18	
Median (IQR)	1.5 (0-10)	5 (0-10)	5 (0-10)	
Education				
<5 years	72 (51.4)	61 (41.2)	133 (46.2)	0.082^ⱡ^
≥5 years	68 (48.6)	87 (58.8)	155 (53.8)	
Total	140 (100)	148 (100)	288 (100)	
Addiction				
Gutka	2 (100)	0 (0)	2 (3.2)	0.832^ǂ^
Pan	16 (55.2)	13 (44.8)	29 (46.8)	
Beetle nut	17 (54.8)	14 (45.2)	31 (50)	
Cigarette	5 (55.6)	4 (44.4)	9 (14.5)	
Naswar	3 (75)	1 (25)	4 (6.5)	
Others	1 (50)	1 (50)	2 (3.2)	
Duration of Diabetes (years)				
Mean ± SD	7.4 ± 7.3	9 ± 7.2	8.2 ± 7.3	0.017^*ꝉ^
Min-Max	2mon-30Y	1mon-30Y	1mon-30y	
Median (IQR)	4.5 (2-10.8)	7 (3-15)	5 (2-12)	
Durations of diabetes				
<3 years	46 (32.9)	32 (21.6)b	78 (27.1)	0.032^*Ɨ^
≥3 years	94 (67.1)	116 (78.4)b	210 (72.9)	
Total	140 (100)	148 (100)	288 (100)	
Duration of diabetes treatment				
Mean ± SD	4.9 ± 5.8	6.4 ± 6.2	10.7 ± 6.2	0.022^*ꝉ^
Min-Max	1.6w-29y	0-29	0-25	
Median (IQR)	3 (1-7)	4.3 (1.5-10)	10 (6-16)	
Duration of diabetes treatment categories				
<3 years	66 (47.1)	47 (31.8)b	113 (39.2)	0.008^*ǂ^
≥3 years	74 (52.9)	101 (68.2)b	175 (60.8)	
Total	140 (100)	148 (100)	288 (100)	
Medication for diabetes				
Oral only	96 (69.1)	81 (54.7)	177 (61.7)	0.041^*ⱡ^
Insulin only	21 (15.1)	30 (20.3)	51 (17.8)	
Both	22 (15.8)	37 (25)	59 (20.6)	
Total	139 (100)	148 (100)	287 (100)	
Comorbids				
HTN	98 (51.3)	93 (48.7)	191 (81.6)	0.180^ǂ^
IHD	16 (41)	23 (59)	39 (16.7)	
Dyslipidemia	74 (49)	77 (51)	151 (64.5)	
Other	20 (39.2)	31 (60.8)	51 (21.8)	
Diabetic related complications				
IHD	10 (34.5)	19 (65.5)	29 (13.6)	0.010^*ǂ^
CVA	4 (36.4)	7 (63.6)	11 (5.1)	
Kidney disease	20 (46.5)	23 (53.5)	43 (20.1)	
Eye damage	59 (41.5)	83 (58.5)	142 (66.4)	
Delayed wound healing	11 (78.6)	3 (21.4)	14 (6.5)	
Foot complications	45 (35.7)	81 (64.3)	126 (58.9)	
Other	17 (53.1)	15 (46.9)	32 (15)	
Depression (PHQ9)				
Minimal/none	23 (16.4)	30 (20.3)	53 (18.4)	0.016^*ǂ^
Mild	48 (34.3)	33 (22.3)	81 (28.1)	
Mderate	37 (26.4)	30 (20.3)	67 (23.3)	
Moderately severe	26 (18.6)	37 (25)	63 (21.9)	
Severe	6 (4.3)	18 (12.2)	24 (8.3)	
Total	140 (100)	148 (100)	288 (100)	
*P-value<0.05, **P-value<0.0001, ꝉ Mann Witney U test,ɫ Independent sample T-test, ƪ Fisher-exact test, ǂ Chi-square test				

Also, it was observed that a higher proportion of patients on oral hypoglycemic agents had inadequate diabetes-related self-care activities in comparison to others (p=0.041, Table 2). Moreover, a larger number of patients with inadequate diabetes-related self-care activities had delayed wound healing while other complications were found to be more in patients with adequate diabetes-related self-care activities (p=0.010, Table 2).

Additionally, a U-shaped relationship was observed between the levels of depression and diabetes-related self-care activities. It was found that a significantly higher proportion of patients who have had mild to moderate depression had inadequate diabetes-related self-care activities in comparison to other groups (p=0.007, Table 2). Interestingly, a greater number of moderately severe to severely depressed patients were found to have adequate diabetes-related self-care activities (Table 2).

## Discussion

Globally, diabetes is considered a serious public health problem. The increasing occurrence of diabetes in both developed and developing states not only increases the financial burden but also accounts for morbidity and lifelong disabilities [18]. The major component of diabetes management is to prevent complications that involve various aspects of understanding disease pathogenesis and progression. Apart from the medication, patient-led diabetes-related self-care activities (DRSCA) play a key role in diabetes management [9].

In this study, we found that two-thirds of the patients had poor glycemic control. This was comparable to findings from other parts of Pakistan reporting 60% of the patients with uncontrolled glycemia [19-20]. The high prevalence of poor glycemic control has also been observed in Middle East countries, i.e. 69% in UAE, 65.1% in Jordan, 65% in Oman, and 78.8% in Kuwait [21], indicating that uncontrolled glycemic control is emerging as an endemic condition globally.

Literature reported the association of various factors, such as gender, employment status, addiction status, treatment of diabetes, its associated complications, and diabetes-related self-care activities with glycemic control [22-23]. However, we were unable to establish a relationship between gender, employment status, education, addiction status, income, and BMI with glycemic control.

However, an inverse relationship between the duration of diabetes and its treatment was observed with glycemic control. It was found that a higher proportion of patients with a duration of illness and treatment ≥3 years had uncontrolled sugar levels in comparison to the others. This inverse relationship is also supported by recent studies [24-25]. This may be due to the progressive loss of β-cells causing impaired insulin secretion and increasing insulin resistance [25].

Interestingly, we even didn’t find an association of diabetes-related self-care activities with glycemic control. The possible reason could be the family support system. Pakistan has a strong family system and family support is valued a lot and plays a significant role in the livings of the family members [26]. It is known that diabetic patients become aggressive with the progression of the disease and lifelong treatment [27]. Family members and relatives are the ones who can tolerate and handle such a type of aggression [27]. Asian families are known for being patient, tolerant and responsible amongst the members regardless of the intensity of the hardship [27]. It might be possible that such patients are being taken care of by their family members, as we have an Asian culture of the joint family system. It has also been reported in another study done in Bangladesh that family offers emotional and economic support, safe food, ensure strict follow-up regimen, improve the battle against anxiety and even reduce deleterious effects of stress on patients [28].

Furthermore, results revealed that a higher proportion of patients who have had an illness and are getting treatment for ≥ 3 years had adequate diabetes-related self-care activities. This demonstrates that patients with a longer duration of diabetes were more compliant with self-care activities. This result of the association between duration and better self-care activities is consistent with the study conducted in Ethiopia, which found that a longer duration leads to improved awareness of the disease, which, in turn, is linked to increased self-efficacy [29].

In our study, treatment modality was also associated with the DSMQ score. A higher proportion of patients who were on oral hypoglycemic agents had inadequate diabetes-related self-care activities. The result is similar to the study conducted in Ethiopia that also found treatment modality as a significant predictor of self-care [30]. Also, our study revealed delayed wound healing in patients with inadequate diabetes-related self-care activities in comparison to the other group. This result was expected, as wound healing requires vigilant monitoring and self-care.

Additionally, we found a U-shaped relationship of severity of depression with diabetes-related self-care activities. It was observed that mild to moderately depressed patients had a higher proportion of inadequate diabetes-related self-care activities. Again, the only reason for this U-shaped relationship could be family support. Severely depressed patients have significant behavioral changes and other psychotic symptoms due to which they might be taken care of by their families more in comparison to mild to moderately depressed patients, whereas normal patients are able to manage the disease themselves. Further studies should be done to establish the relationship between the severity of depression and diabetes-related self-care activities.

Limitations

The study didn’t include any questions regarding family and social support, which could have an impact on self-care activities for diabetes management. Second, the study was cross-sectional so causal inferences and temporality could not be developed. Third, this is a single-center, hospital-based study. Prospective community-based studies should be conducted in the future to study the phenomenon more deeply and accurately.

## Conclusions

It was found that a longer duration of illness was associated with poor glycemic control but adequate diabetes-related self-care activities. Furthermore, diabetes-related self-care activities were found to have no impact on glycemic control.
